# Targeting circular RNAs as a therapeutic approach: current strategies and challenges

**DOI:** 10.1038/s41392-021-00569-5

**Published:** 2021-05-21

**Authors:** Alina T. He, Jinglei Liu, Feiya Li, Burton B. Yang

**Affiliations:** 1 Sunnybrook Research Institute, Toronto, ON Canada; 2 Department of Bioinformatics, ATCGene Inc, Guangzhou, China; 3 Department of Laboratory Medicine and Pathobiology, University of Toronto, Toronto, ON Canada

**Keywords:** Gene delivery, Gene therapy

## Abstract

Significant progress has been made in circular RNA (circRNA) research in recent years. Increasing evidence suggests that circRNAs play important roles in many cellular processes, and their dysregulation is implicated in the pathogenesis of various diseases. CircRNAs are highly stable and usually expressed in a tissue- or cell type-specific manner. Therefore, they are currently being explored as potential therapeutic targets. Gain-of-function and loss-of-function approaches are typically performed using circRNA expression plasmids and RNA interference-based strategies, respectively. These strategies have limitations that can be mitigated using nanoparticle and exosome delivery systems. Furthermore, recent developments show that the cre-lox system can be used to knockdown circRNAs in a cell-specific manner. While still in the early stages of development, the CRISPR/Cas13 system has shown promise in knocking down circRNAs with high specificity and efficiency. In this review, we describe circRNA properties and functions and highlight their significance in disease. We summarize strategies that can be used to overexpress or knockdown circRNAs as a therapeutic approach. Lastly, we discuss major challenges and propose future directions for the development of circRNA-based therapeutics.

## Introduction

Circular RNAs (circRNAs) are covalently closed loop structures that lack free 3’ and 5’ ends.^1^ CircRNAs have long been understudied, and their regulatory potential has yet to be fully uncovered. Initially, circRNAs were regarded as rare isoforms produced as a consequence of splicing errors. However, with the development of bioinformatics and high-throughput sequencing, it is now well documented that circRNAs are abundantly expressed and highly conserved across species.^2–7^ The first circRNA function that was elucidated was acting as a sponge for microRNA to upregulate the expression of their target mRNA.^8^ Following this discovery, studies found that circRNAs perform many other regulatory functions, including exerting transcriptional and translational control,^9–12^ sequestering and translocating proteins,^13–15^ facilitating interactions between proteins,^16–19^ and translating to proteins.^20–22^

Dysregulation of circRNAs has been implicated in a wide variety of diseases, especially cancers, cardiovascular diseases, and neurological diseases.^23^ Given that circRNAs are highly stable and usually exhibit tissue- or cell type-specific expression,^24–26^ they could potentially serve as effective therapeutic targets. CircRNAs are typically overexpressed using expression plasmids and knocked down using RNA interference (RNAi)-based strategies. RNAi molecules have many limitations, including their instability, lack of cell-specificity, low intracellular entry, immune system activation, and other off-target effects.^27–29^ Using nanoparticles or exosomes as delivery systems for these molecules can improve their stability, intracellular entry, and immunogenicity.^30–32^ Recently, the cre-lox system was used to knockdown circRNAs in specific cells.^33,34^ Furthermore, CRISPR technology and Cas13 systems in particular have demonstrated great potential in knocking down circRNAs in a specific and robust manner.^35,36^

In this review, we discuss circRNA properties and functions, followed by their significance in diseases. We describe approaches that can be used to target circRNAs and address their therapeutic potential. Finally, we identify key challenges in developing circRNA-based therapeutics and propose future directions.

## Biogenesis and properties of circRNAs

**Fig. 1:**
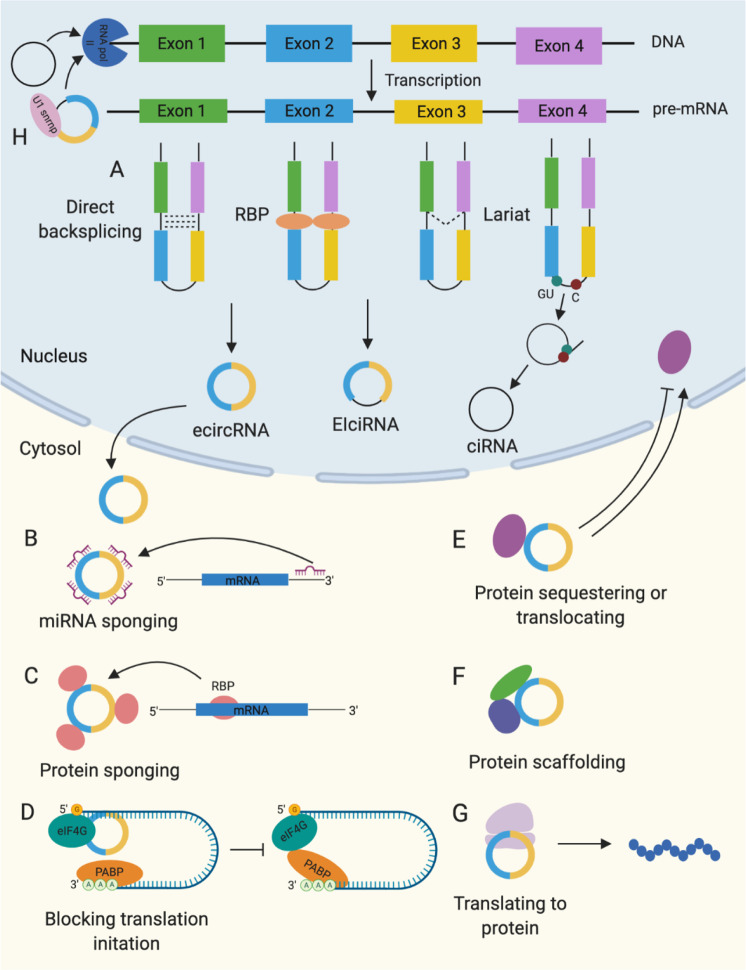
Biogenesis and functional mechanisms of circular RNAs (circRNAs). **A** Back-splicing driven by the pairing of intronic complementary sequences, RNA-binding protein (RBP), or lariat structure containing skipped exons or introns. **B** Sponging microRNA (miRNA) to decrease their availability to bind target mRNA. **C** Sponging RNA-binding protein (RBP) to decrease their availability to bind target mRNA. **D** Interacting with eukaryotic translation initiation factor 4 G (eIF4G), poly(A)-binding protein (PABP), and cognate mRNA to disrupt the assembly of the translation initiation machinery. **E** Translocating proteins to the nucleus or sequestering them in the cytosol. **F** Facilitating interactions between specific proteins. **G** Translating to protein in a cap-independent manner. **H** Exon-intron circRNAs (EIcircRNAs) can form a complex with the U1 small nuclear ribonucleoprotein (U1 snRNP) that binds RNA polymerase II (RNA pol II) to enhance transcription of parental genes. Intronic circRNAs (ciRNAs) can interact with elongating RNA pol II complex to enhance transcription

CircRNAs can be categorized as exonic (ecircRNA), exon-intron (EIcircRNA), or intronic (ciRNA) circRNAs. The majority of circRNAs are ecircRNAs, which are predominantly located in the cytoplasm.^2^ In contrast, EIcircRNAs and ciRNAs are usually located in the nucleus.^9,10^ There are three proposed models of circRNA biogenesis: direct back-splicing, RNA-binding protein-mediated circularization, and lariat-driven circularization, which are depicted in Fig. 1. During back-splicing, a downstream splice donor is joined with an upstream splice acceptor. In direct back-splicing, the splice sites are brought in close proximity by the complementary base pairing of inverted repeats in introns flanking the circularized exons.^37,38^ In addition, RNA-binding protein (RBP) can bind specific motifs in the flanking introns to promote circularization.^38–40^ Several splicing factors, including Quaking^39^ and muscleblind,^40^ have been shown to facilitate circRNA production. Heterogeneous nuclear ribonucleoprotein (hnRNP) and serine-arginine (SR) proteins have also been found to work in conjunction with intronic repeats to regulate circRNA biogenesis in a combinatorial manner.^38^ Furthermore, nuclear factor NF90 and isoform NF110 strongly promoted back-splicing by stabilizing the pairing of intronic complementary sequences.^41^ After circularization, the intervening intron is removed to form ecircRNAs or retained to form EIcircRNAs. Alternatively, circRNAs can be generated through lariat-driven circularization. Exon-skipping during pre-mRNA processing produces a lariat structure containing the skipped exons.^42^ The lariat structure can then undergo internal splicing to form ecircRNAs or EIcircRNAs. The formation of ciRNAs is thought to occur from lariats produced from introns removed during pre-mRNA splicing. These structures are usually debranched and degraded, but those with a 7 nt GU-rich element near the 5’ splice site and an 11 nt C-rich element near the branch-point site are spared and can subsequently produce ciRNAs.^10^

Aberrant regulation of circRNA biogenesis may play a role in disease. In general, circRNA biogenesis uses canonical splice sites, and thus back-splicing can compete with linear splicing of mRNA. Under physiological conditions, back-splicing is usually less efficient than linear splicing.^43^ However, depleting the activity of core spliceosomal components resulted in increased expression of circRNAs, while expression of their associated linear mRNAs decreased. The balance between back-splicing and linear splicing could also be altered due to RBPs that facilitate circularization. Dysregulation in Quaking^44^ and muscleblind^45^ splicing factors have been implicated in a wide array of pathological conditions. It is also possible that mutations in the intronic repeats could affect circularization.^46^

Recently, a large-scale transcriptome analysis was performed in the human, macaque, and mouse.^25^ It was estimated that in humans, 61% of genes produce both circular and linear transcripts. CircRNAs are generally transcribed at low levels compared to mRNA, but some are highly expressed.^47,48^ Some genes produce a dominant circRNA, whose expression is significantly enhanced compared to other circRNAs produced from the same gene.^25^ It was reported that 64% of circRNAs are only expressed in one type of tissue. Dominant circRNAs were found to be less tissue specific. Still, for genes expressed in two or more tissues, only 18% produced a circRNA that was dominant across different tissues. Importantly, it was shown that the association between circRNA expression and parent gene expression is weak, suggesting that circRNAs are largely independently regulated and not mere byproducts of aberrant splicing.^25^ Furthermore, circRNAs are resistant to digestion by exonucleases due to their closed loop structure and are thus more stable than linear RNAs.^49,50^ It was reported that the median half-life of circRNAs in mammary cells is 18.8–23.7 h compared to 4.0–7.4 h for their corresponding linear mRNAs.^24^ Altogether, the expression pattern and stability of circRNAs suggest that they likely hold functional significance.

## Functional mechanisms of circRNAs

To date, various circRNA functions have been elucidated. Although microRNA sponging is the most well-known function, circRNAs perform many other functions and exert widespread regulatory effects. We provide a brief overview of circRNA functions in Fig. 1 and summarize their mechanisms below.

### Acting as miRNA sponges

The most extensively reported function of circRNAs is microRNA (miRNA) sponging.^51–54^ miRNAs are small noncoding RNAs that bind to target mRNAs and typically induce mRNA degradation or translational repression.^55–59^ Many circRNAs have been found to extensively bind miRNAs, decreasing their availability and thereby upregulating the expression of their target mRNAs.^8,49,60^ The first cases of miRNA sponging were discovered for CDR1as, with over 70 conserved target sites for miR-7, and circSry, with 16 binding sites for miR-138.^8^ CircRNAs functioning as a miRNA sponge continue to be frequently documented. However, studies that analyzed thousands of circRNAs found that most contain a smaller number of miRNA binding sites and do not have other properties of effective miRNA sponges.^37,48^ These findings suggest that the majority of circRNAs do not act as miRNA sponges, and many studies have revealed other functions.

### Regulating transcription and translation

CircRNAs have been shown to exert transcriptional and translational control, especially over their parent genes. Although most circRNA functions have been described for ecircRNAs, a few studies have uncovered regulatory functions of EIcircRNAs and ciRNAs in the nucleus. EIcircRNAs have been proposed to interact with U1 small nuclear ribonucleoprotein (U1 snRNP) in a complex, which in turn interacts with RNA polymerase II to promote the transcription of their parent genes.^9^ Similarly, it has been suggested that ciRNAs associate with the elongating RNA polymerase II complex to enhance transcription of their parent genes.^10^ Recently, a ciRNA derived from the insulin gene was found to interact with RBP TDP-43 and exert transcriptional control over various genes necessary for insulin secretion.^61^ In terms of translational control, it has been shown that circPABPN1 extensively binds to RBP HuR to reduce HuR binding to PABPN1 mRNA, ultimately suppressing its translation.^11^ We recently showed for the first time that a circRNA can directly bind its parent mRNA to regulate its translation. CircYap was found to bind Yap mRNA and translation initiation proteins eIF4G and PABP.^12^ Overexpressing circYap disrupted the interaction between PABP on the 3’-tail with eIF4G on the 5’-cap of Yap mRNA, thereby blocking translation. Interaction sites for circYap-Yap mRNA, -PABP, and -eIF4G were identified in silico. Mutating any of the three sites rescued the translational repression. These findings suggest that circYap binds to Yap mRNA, eIFG4, and PABP to disrupt the assembly of the translation initiation machinery.^12^ Previous studies did not find interactions between circRNAs and their corresponding linear mRNAs, suggesting that this function may not be common. The interaction between circRNA and mRNA is likely dependent on their primary sequences and tertiary structures. In silico analysis can be performed to predict their interaction sites.

### Sequestering and translocating proteins

CircRNAs can sequester proteins in or translocate proteins between subcellular compartments. CircFoxo3 was shown to bind several proteins, ID-1, E2F1, HIF1α, and FAK that exert protective effects against cardiac senescence, promoting their retention in the cytoplasm and suppressing their downstream activity.^62^ In contrast, circAmotl1 facilitated the nuclear translocation of several proteins: c-myc, an oncogenic transcription factor,^63^ protein kinase B/AKT, which protected against cardiomyopathy,^13^ and STAT3, a transcription factor that contributed to skin wound repair,^14^ ultimately promoting their respective functions. The mechanisms that allow circRNAs to influence protein trafficking are currently unknown.

### Facilitating interactions between proteins

Some circRNAs have been found to act as protein scaffolds and facilitate interactions between proteins. CircFoxo3 was shown to bind CDK2 and p21, which simultaneously facilitated p21-mediated suppression of CDK2 activity and prevented CDK2 from binding cyclin E, arresting cell cycle progression in the G1 phase.^16^ CircFoxo3 was also shown to bind p53 and MDM2 to form a complex that facilitated MDM2-induced p53 ubiquitination and relieved MDM2-induced Foxo3 ubiquitination, leading to increased PUMA expression and tumor cell apoptosis.^17^ These studies and others^18,19^ suggest that circRNAs frequently interact with proteins. Bioinformatics analysis of nucleotide sequences concluded that circRNAs have a lower RBP binding density compared to linear mRNA.^64^ However, analysis of circRNA nucleotide sequences alone may not be accurate in predicting circRNA-protein interactions, as their three-dimensional structures heavily influence their protein binding capacity.^63^

### Translating to proteins

Although circRNAs are generally noncoding, several studies have provided evidence that some circRNAs can be translated into proteins. It was found that the circular transcript of the scRYMV RNA virusoid has the ability to translate into a protein.^20^ Later, it was reported that circMbl3^21^ and circZNF609^22^ can translate into proteins in a cap-independent and internal ribosome entry site (IRES)-dependent manner. In addition, the RNA base modification N^6^-methyladenosine (m^6^A) was found to drive efficient translation initiation of circRNAs.^65^ The functional significance of many circRNA-derived proteins has yet to be determined, although some have been implicated in cancers.^66^

## Targeting circRNAs in disease

The dysregulation of circRNA expression has been implicated in a wide variety of diseases, especially cancers, cardiovascular diseases, and neurological disorders.^23,67–70^ Many studies have described functional roles for circRNAs in promoting these diseases or exerting protective effects against them.^67^ In this section, we present recent studies that used gain-of-function and loss-of-function approaches to demonstrate the therapeutic potential of targeting circRNAs (Table 1).

**Table 1:** Dysregulated circRNAs that have been targeted in diseases

Related disease	circRNA ID	Dysregulation	Reference
*Cancer*			
Triple negative breast cancer (TNBC)	circAGFG1	Up	^74^
Breast cancer	circDnmt1	Up	^15^
Triple negative breast cancer (TNBC)	circHER2	Up	^75^
Triple negative breast cancer (TNBC)	circTADA2A-E6	Down	^76^
Breast cancer	Hsa_circ_0025202	Down	^77^
Hepatocellular carcinoma (HCC)	circβ-catenin	Up	^79^
Hepatocellular carcinoma (HCC)	circRNA-104718	Up	^80^
Hepatocellular carcinoma (HCC)	circTRIM33-12	Down	^81^
Non-small cell lung cancer (NSCLC)	circRNA 100146	Up	^82^
Non-small cell lung cancer (NSCLC)	circPTPRA	Down	^83^
Gastric cancer (GC)	circCACTIN	Up	^84^
Gastric cancer (GC)	circPSMC3	Down	^85^
Gastric cancer (GC)	circHuR	Down	^86^
Gastric cancer (GC) Colon cancer Prostate cancer Neuroblastoma	circAGO2 (hsa_circ_0135889)	Up	^87^
Neuroblastoma	circCUX1 (hsa_circ_0132813)	Up	^88^
Colorectal cancer (CRC)	circLONP2 (has_circ_0008558)	Up	^89^
*Cardiovascular disease*			
Myocardial infarction (MI)	circNfix	Down	^92^
Myocardial infarction (MI)	circTtc3	Up	^93^
Cardiac fibrosis (CF)	circHIPK3	Up	^94^
Cardiac fibrosis (CF)	circNFIB	Down	^95^
Cardiac fibrosis (CF)	circYap	Down	^96^
Doxorubicin–induced cardiomyopathy	circFoxo3	Up	^62^
Ischemia/reperfusion (I/R)	ACR	Down	^97^
Cardiac hypertrophy	circSlc8a1	Up	^98^
Intimal hyperplasia	circ_Lrp6	Up	^99^
*Central nervous system disease*			
Alzheimer’s disease (AD)	CDR1as/ ciRS-7	Down	^102^
Acute ischemic stroke (AIS)	circHECTD1	Up	^104^
Acute ischemic stroke (AIS)	circTLK1	Up	^105^
Acute ischemic stroke (AIS)	circSCMH1	Down	^106^
Neuropathic pain	circAnks1a	Up	^107^
Diabetes-related neuropathic pain	circHIPK3	Up	^108^
Neuropathic pain	circRNA.2837	Down	^109^
Depression	circHIPK2	Up	^111^
Blood–brain barrier (BBB) damage Acute ischemic stroke (AIS) Parkinson’s disease (PD)	circDLGAP4	Down	^113,114^
*Other diseases*			
Hepatic fibrosis	circPWWP2a (hsa_circ_0074837)	Up	^116^
Pulmonary fibrosis	circHIPK3	Up	^117^
Diabetes-related retinal vascular dysfunction	circHIPK3	Up	^118^
Retinal vascular dysfunction Glaucoma	cZNF609 (mmu_circ_0001797)	Up	^119,120^
Diabetes-related retinal vascular dysfunction	cPWWP2a	Up	^121^
Osteoarthritis (OA)	circRNA.33186	Up	^122^
Osteoarthritis (OA)	circSERPINE2 (has_circ_0008365)	Down	^123^
Intervertebral disc degeneration (IDD)	circRNA_104670	Up	^124^
Intervertebral disc degeneration (IDD)	circVMA21	Down	^125^
Bone nonunion	has_circ_0074834	Down	^126^

### Targeting circRNAs in cancer

The expression profile of circRNAs in human cancers is diverse. A recent pan-cancer study performed high-throughput exome capture RNA sequencing on more than 2000 patient samples to provide a global perspective on circRNA expression in cancers.^71^ Results showed that the expression profile of circRNAs in different cancers are distinct, and the expression of a given circRNA across cancer types is significantly different. These findings, taken together with the stability of circRNAs, suggest that they can be used as potential cancer biomarkers.^72^ A recent study provided strong evidence that circRNAs have functional roles in cancer that are independent of their linear counterparts. A high-throughput shRNA-based screening was performed to evaluate the importance of specific circRNAs in prostate cancer.^73^ This analysis identified 171 circRNAs as essential for cell proliferation. Interestingly, for ~90% of them, their corresponding linear transcripts were not considered essential. Many additional functional studies have uncovered oncogenic or antitumor functions of circRNAs. These circRNAs could serve as potential therapeutic targets. Below, we highlight some examples of circRNAs that were targeted in different types of cancers.

#### Breast cancer

CircAGFG1 was upregulated in triple negative breast cancer (TNBC) tissues and played oncogenic roles through miR-195-5p sponging.^74^ Short hairpin RNA (shRNA) targeting circAGFG1 suppressed cell proliferation, migration, and invasion and increased apoptosis in vitro. Furthermore, tumor growth, angiogenesis, and metastasis were reduced in vivo.^74^ CircDnmt1 was also significantly upregulated in breast cancer. CircDnmt1 facilitated the nuclear translocation of p53 and AUF1 proteins, resulting in tumor growth and cellular autophagy.^15^ Delivery of gold nanoparticles (PEG-AuNPs) conjugated with short interfering RNA (siRNA) targeting circDnmt1 suppressed these effects in mice. More recently, circHER2 was found to be highly expressed in TNBC samples and encoded a novel 103aa peptide, HER2–103, and played a role in tumorigenesis.^75^ This peptide shared most of its amino acid sequence with the HER2 CR1 domain, which can be antagonized by Pertuzumab. Accordingly, Pertuzumab significantly reduced the tumorigenicity of circHER2 and HER2–103 expressing cells in vivo.^75^ In contrast, circTADA2A-E6 was downregulated in TNBC tissues and acted as a miR-203a-3p sponge.^76^ Overexpression of circTADA2A-E6 suppressed cell proliferation, migration, and invasion in vitro. Hsa_circ_0025202 was also significantly downregulated in breast cancer and functioned as a sponge for miR-182-5p.^77^ Its overexpression not only had antitumor effects, but it also enhanced breast cancer cell sensitivity to tamoxifen in vitro and in vivo. Expression of circSka3 is highly upregulated in breast cancer patients. This circRNA forms invadopodia by binding to Tks5 and Integrin β1, markers of invadopodia, resulting in enhanced tumor invasion and metastasis. Silencing circSka3 with siRNA or inhibiting its interactions to Tks5 and Integrin β1 using a blocking oligonucleotide reversed its effects.^78^ Thus, circSka3 could serve as a potential target to block breast cancer progression.

#### Liver cancer

Circβ-catenin was upregulated in liver cancer tissues.^79^ It was found to translate into a novel β-catenin isoform that acts as a decoy for Gsk3β, ultimately promoting Wnt signaling. shRNA targeting circβ-catenin suppressed cell proliferation, migration, and invasion in vitro and attenuated tumorigenesis and metastasis in vivo.^79^ In addition, circRNA-104718 was upregulated in hepatocellular carcinoma (HCC) and was found to sponge miR-218-5p.^80^ circRNA-104718 shRNA resulted in similar antitumor effects. In contrast, circTRIM33-12 was downregulated in HCC tissues and functioned as a sponge for miR-191.^81^ Its overexpression inhibited cell proliferation, invasion, and migration in vitro and suppressed tumor growth and metastasis in vivo.

#### Lung cancer

CircRNA 100146 was upregulated in non-small cell lung cancer (NSCLC) tissues.^82^ This circRNA was found to bind multiple splicing factor family SF3 proteins, as well as miR-361-3p and miR-615-5p. siRNA against circRNA 100146 reduced cell proliferation, migration, and invasion and increased apoptosis in vitro, as well as inhibited tumor growth in vivo.^82^ Conversely, circPTPRA was downregulated in NSCLC tissues and exerted tumor-suppressing effects by sponging miR-96-5p.^83^ Overexpression of circPTPRA inhibited cell migration, invasion, and epithelial-to-mesenchymal transitioning (EMT) in vitro and tumor metastasis in vivo.

#### Gastric cancer

CircCACTIN was upregulated in gastric cancer (GC) tissues and was found to sponge miRNA-331-3p.^84^ siRNA targeting circCACTIN reduced cell proliferation, migration, and invasion in vitro and inhibited tumor growth and EMT in vivo. In contrast, circPSMC3 was downregulated in GC tissues and played antitumor roles via miR-296-5p sponging.^85^ Overexpression of this circRNA suppressed cell proliferation, migration, and invasion in vitro, as well as tumor growth and metastasis in vivo. CircHuR was also downregulated in GC, and its overexpression resulted in similar antitumor effects.^86^ Mechanistically, circHuR was shown to interact with the CNBP transcription factor, which prevents CNBP binding to the HuR promoter, thereby reducing HuR expression.

#### Other cancers

CircAGO2 was found to be activated by HuR protein and upregulated in many cancer tissues.^87^ Activation of circAGO2 resulted in increased proliferation, invasion, and metastasis in vitro and in vivo. shRNA targeting circAGO2 repressed tumorigenesis in mice. The effects of circAGO2 could also be repressed by blocking circAGO2 and HuR interaction through delivery of a HuR inhibitory peptide.^87^ In neuroblastoma (NB) tissue, circCUX1 was highly upregulated and promoted cell proliferation, migration, and invasion via sponging miR-16-5p.^88^ Delivery of circCUX1 shRNA reduced tumor growth in mice. Antisense oligonucleotides have also been used to silence upregulated circRNAs. CircLONP2 directly interacted with DDX1 and was upregulated in metastasis-initiating cells in colorectal cancer (CRC), promoting migration and invasion. Antisense oligonucleotide-mediated silencing of circLONP2 in vivo suppressed CRC metastasis.^89^

### Targeting circRNAs in cardiovascular disease

CircRNAs are abundant in the human heart, and many of them are cardiac specific.^90^ Numerous studies have elucidated functional roles of circRNAs in aggravating cardiovascular diseases or exerting cardio-protective effects.^91^ Here, we describe some studies that targeted circRNAs in various cardiovascular diseases.

#### Myocardial infarction

CircNfix was highly expressed in adult cardiomyocytes in mice, rats, and humans but was initially downregulated in mice post-myocardial infarction (MI).^92^ This circRNA was reported to sponge miR-214 and upregulate Gsk3β, and also facilitate the interaction between Ybx1 and E3 ubiquitin-protein ligase Nedd4I. shRNA knockdown of circNfix increased cell proliferation and angiogenesis and reduced apoptosis, thereby promoting cardiac regenerative repair post-MI.^92^ Meanwhile, circTtc3 was upregulated in the myocardium of post-MI rats and acted as a sponge for proapoptotic miR-15b.^93^ Overexpression of circTtc3 in cardiomyocytes inhibited apoptosis and exerted protective effects against pathological cardiac remodeling post-MI.

#### Cardiac fibrosis

CircHIPK3 was upregulated in cardiac fibroblasts and mouse heart tissues after Angiotensin-II treatment and sponged miR-29b-3p.^94^ CircHIPK3 siRNA reduced proliferation and migration in vitro, and circHIPK3 shRNA attenuated cardiac fibrosis in vivo.^94^ Conversely, circNFIB was downregulated in cardiac fibroblasts treated with TGF-β and heart tissues of mice post-MI.^95^ Overexpression of circNFIB attenuated cardiac fibroblast proliferation in vitro by sponging profibrotic miR-433. Recently, circYap was found to be downregulated in cardiac tissues of patients with cardiac hypertrophy and in a transverse aortic constriction (TAC) mouse model of pressure overload.^96^ Delivery of nanoparticle-conjugated circYap plasmids attenuated cardiac fibrosis and improved heart function by decreasing actin polymerization in TAC mice.

#### Other cardiovascular diseases

CircFoxo3 was highly expressed in hearts of aged patients and mice and correlated with markers of senescence. Silencing circFoxo3 through siRNA inhibited senescence and attenuated Doxorubicin (Dox)-induced cardiomyopathy.^62^ In addition, ACR circRNA expression was significantly downregulated in mouse heart tissues after ischemia/reperfusion (I/R) injury.^97^ ACR sequestered Dnmt3B to prevent Dnmt3B-induced methylation of the Pink1 promoter. Overexpression of ACR reduced cardiomyocyte autophagy and cell death in vitro and attenuated I/R injuries in vivo.^97^ CircSlc8a1 was highly enriched in mouse and human cardiomyocytes.^98^ In a pressure-overload cardiac hypertrophy mouse model, circSlc8a1 was found to sponge miR-133a, known to suppress cardiac hypertrophy. Accordingly, shRNA-mediated knockdown of circSlc8a1 exerted protective effects against cardiac hypertrophy in vivo.^98^ Finally, circ_Lrp6 expression was elevated in mouse and human vascular smooth muscle cells (VSMC) and acted as a sponge for miR-145.^99^ Circ_Lrp6 shRNA suppressed VSMC proliferation and migration in vitro and reduced intimal hyperplasia in the carotid arteries of a mouse model of stenosis.

### Targeting circRNAs in central nervous system (CNS) diseases

CircRNAs are also highly abundant in the brain. Studies have evaluated circRNA expression in various brain regions. It was estimated that roughly 30% of genes transcribed in the human brain produce circRNAs.^100^ We highlight some studies that have targeted circRNAs in central nervous system (CNS) diseases.

#### Neurodegenerative diseases

CDR1as is highly expressed in the brain and has been implicated in neurodegenerative diseases, such as Alzheimer’s disease (AD) and Parkinson’s disease (PD). CDR1as was previously found to be downregulated in the brain of individuals with AD.^101^ It reduced APP and BACE1 levels in a NF‐κB dependent manner. Overexpression of CDR1as decreased the production of amyloid-β (Aβ) peptides, thereby exerting a neuroprotective role.^102^ Furthermore, a circRNA containing the Aβ-coding region of the *APP* gene has been identified in the brain.^103^ It was demonstrated that this circRNA efficiently translated into a novel protein in vitro and in the human brain, which could be subsequently processed into Aβ peptides. Thus, this circRNA represents a potential therapeutic target for AD.

#### Acute ischemic stroke

CircHECTD1 was significantly upregulated in the plasma of acute ischemic stroke (AIS) patients and in a transient middle cerebral artery occlusion (tMCAO) mouse stroke model.^104^ CircHECTD1 sponged miR-142 to upregulate TIPARP and inhibited astrocyte activation via autophagy. siRNA targeting circHECTD1 reduced astrocyte activation and infarction in vivo. Similarly, circTLK1 was upregulated in AIS patients and tMCAO mice and also regulated TIPARP but through sponging miR-335-3p.^105^ shRNA targeting circTLK1 reduced infarction and neurological deficits in vivo. Conversely, circSCMH1 was downregulated in the plasma of AIS patients and photothrombotic stroke mice.^106^ It was found to bind MeCP2 transcription factor, derepressing its target genes. Excitingly, overexpression of circSCMH1 using intravenous injection of rabies virus glycoprotein-circSCMH1-extracellular vesicles (RVG-circSCMH1-EVs) promoted functional recovery after stroke in mice and rhesus monkeys.

#### Neuropathic pain

CircAnks1a was upregulated in the dorsal horn following spinal nerve ligation (SNL) in rats.^107^ It was found to upregulate VEGFB through several mechanisms, resulting in increased excitability of dorsal horn neurons and pain-like behavior following SNL. Spinal injection of circAnks1a siRNA alleviated the pain-like behavior. In addition, circHIPK3 was upregulated in the serum of diabetes patients with neuropathic pain and in the dorsal root of diabetic rats and was found to target miR-124.^108^ CircHIPK3 shRNA significantly attenuated the neuropathic pain in diabetic rats. In contrast, circRNA.2837 was downregulated in a sciatic nerve injury rat model.^109^ It targeted miR-34a and negatively regulated autophagy. Delivery of circRNA.2837 inhibitor to the injured nerve resulted in protective effects by inducing autophagy.

#### Other CNS diseases

CircHIPK2 promoted astrocyte activation through sponging miR-124-2hg and upregulating SIGMAR1.^110^ Delivery of circHIPK2 siRNA into the hippocampus significantly prevented astrocyte activation through regulation of ER stress and autophagy in mice. A more recent study on the role of circHIPK2 in depression showed transplantation of gut microbiota from NLRP3 KO mice alleviated astrocyte dysfunction and depression-like behavior induced by chronic unpredictable stress through inhibition of circHIPK2.^111^ Delivering siRNA targeting circHIPK2 into the hippocampus recovered astrocyte dysfunction and attenuated depression-like behavior. CircHIPK2 shRNA specifically targeting astrocytes in the hippocampus also reversed chronic unpredictable stress-induced astrocyte and behavioral changes. Intravenous delivery of circHIPK2 did not influence its expression in the brain or affect astrocyte function or depression-like behavior. Drug delivery across the blood–brain barrier (BBB) has been a long-standing challenge in the field of neuropharmacology, and this is an issue for the circRNA field to consider as well. Another study showed that circHECW2 regulated the endothelial-mesenchymal transition (EMT) via binding miR-30d and upregulating ATG5.^112^ Delivery of circHECW2 siRNA in the mouse hippocampus efficiently reduced EMT. Given the role of EMT in BBB damage, this may serve as a potential therapeutic approach to improve BBB integrity. Finally, circDLGAP4 was downregulated in the plasma of AIS patients and tMCAO mice and acted as a sponge for miR-143.^113^ Overexpression of circDLGAP4 in tMCAO mice resulted in inhibitory effects on EMT, which led to improved BBB integrity, decreased infarction, and alleviated neurological deficits. A more recent study found circDLGAP4 also exerted neuroprotective effects in Parkinson’s disease through regulation of miR-134-5p and CREB.^114^

### Targeting circRNAs in other diseases

Synthetic circRNAs have been engineered to efficiently sponge miR-122, which is necessary for the life cycle of Hepatitis C Virus (HCV). The delivery of this artificial circRNA inhibited HCV viral protein production in vitro.^115^

CircPWWP2a in hepatic stellate cells was a common downstream target of fibrosis related proteins TGF-β and LPS and promoted liver fibrosis through sponging miR-203 and miR-223.^116^ circPWWP2a shRNA effectively reduced fibrosis hallmarks in a liver fibrosis mouse model. In addition, circHIPK3 was upregulated in patients with idiopathic pulmonary fibrosis and in bleomycin-induced pulmonary fibrosis mice and functioned as a miR-338-3p sponge.^117^ Intratracheal delivery of circHIPK3 shRNA efficiently regulated fibroblast to myofibroblast transitioning and inhibited fibroblast proliferation in vitro and in vivo. In a wound healing mouse model, overexpression of circAmotl1 using gold nanoparticles accelerated wound healing by promoting Stat3 expression and nuclear translocation, Dnmt3a expression, and fibronectin levels.^14^

In addition to diabetes-related neuropathic pain and pulmonary fibrosis, circHIPK3 was also upregulated in diabetic retinas and retinal endothelial cells following diabetes mellitus-related stress inducers and acted as a sponge for miR-30a-3p.^118^ Intravitreal injection of circHIPK3 shRNA effectively relieved retinal vascular dysfunction. Furthermore, cZNF609 sponged miR-615 and has been implicated in retinal vascular dysfunction,^119^ as well as retinal neurodegeneration.^120^ Intravitreal delivery of cZNF609 shRNA reduced retinal vascular damage and constrained overactivation of angiogenesis in a diabetic retinopathy mouse model. CZNF609 shRNA also conferred protective effects on retinal ganglion cells in a glaucoma rat model. Lastly, cPWWP2a has also been implicated in diabetes-related retinopathy.^121^ It was upregulated in pericytes but not endothelial cells and functioned as a miR-579 sponge. In vitro work suggests that cPWWP2a could be transported by exosomes to indirectly exert its regulatory effects on endothelial cells. Its overexpression in vivo alleviated diabetes-induced retinal vascular dysfunction.

In musculoskeletal disease, circRNA.33186 was highly expressed in a osteoarthritis (OA) mouse model.^122^ It was shown to inhibit miR-127-5p and increase MMP-13 levels, contributing to OA development. Silencing circRNA.33186 by siRNA intra-articular injection efficiently alleviated OA outcomes. In contrast, circSERPINE2 expression was decreased in OA cartilage tissues.^123^ It acted as a sponge for miR-1271-5p, and its overexpression in a rabbit OA model resulted in improved cartilage surface and reduced OA. Furthermore, circRNA_104670 was upregulated in intervertebral disc degeneration (IDD) tissues.^124^ It was determined that circRNA_104670 bound to miR-17-3p, thereby upregulating MMP2. Delivery of siRNA targeting circRNA_104670 alleviated the IDD process in mice. On the other hand, circVMA21 was downregulated in IDD and acted as a sponge for miR-200c, which targeted XIAP.^125^ Overexpression of circVMA21 similarly protected the intervertebral disc from degeneration. Finally, in bone marrow mesenchymal stem cells (BMSCs) collected from bone nonunion patients, has_circ_0074834 was significantly downregulated and was found to regulate ZEB1 and VEGF via miR-942-5p.^126^ Its overexpression promoted osteogenic differentiation of BMSCs in vivo and facilitated bone regeneration in a bone defect mouse model.

## Strategies to target circRNAs

**Fig. 2:**
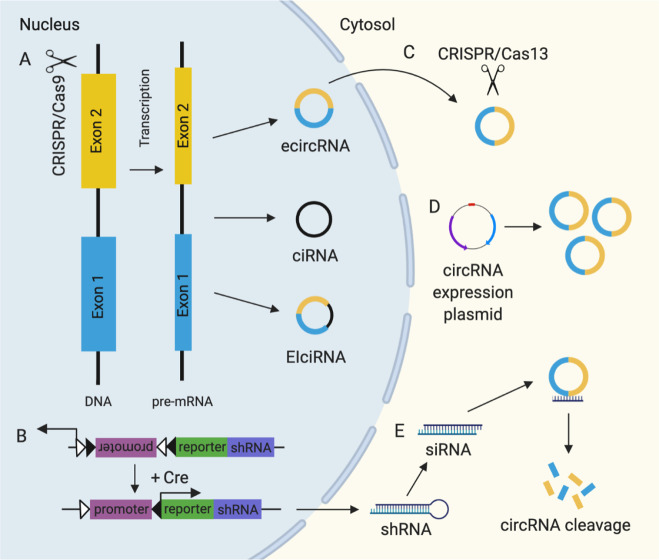
Strategies used to study circular RNA (circRNA). **A** CRISPR/Cas9-mediated circRNA knockout via removal of intronic complementary sequence flanking circularized exon involved in circRNA biogenesis. This system has also been used to target the entire gene locus and a transcription factor to knockout and knockdown circRNA, respectively (not shown). **B** Conditional circRNA knockdown mediated by a cre-dependent short hairpin RNA (shRNA), which is subsequently processed into short interfering RNA (siRNA) to induce circRNA cleavage. **C** CRISPR/Cas13-mediated circRNA knockdown directly targets the back-splice junction of circRNAs to induce circRNA cleavage. **D** CircRNA expression plasmid leads to circRNA overexpression. **E** siRNA/shRNA targeting the back-splice junction of circRNAs induces circRNA cleavage

**Fig. 3:**
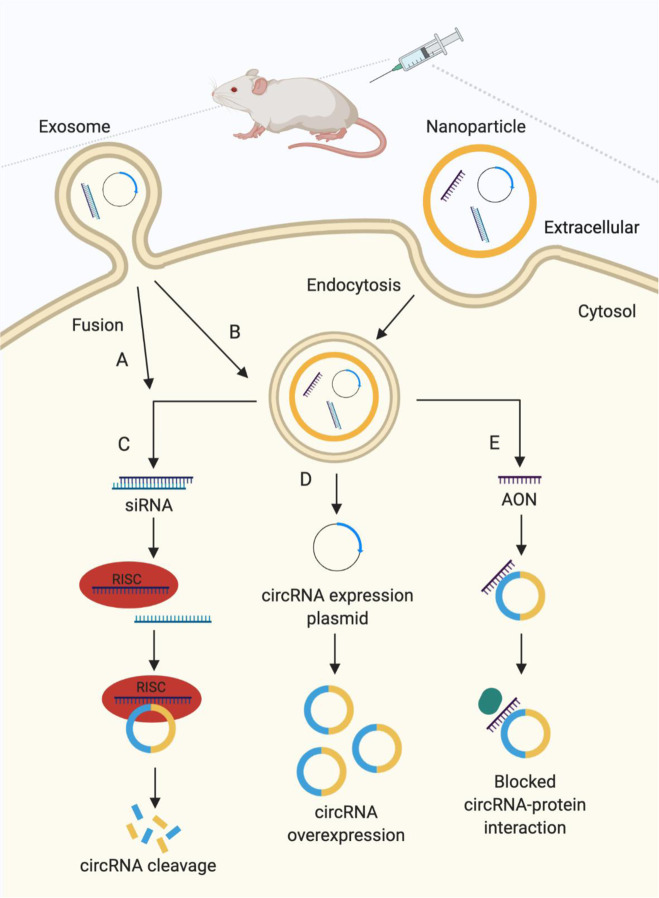
Strategies used to target circular RNAs (circRNAs) as a therapeutic approach in vivo. **A,** **B** Exosome-mediated delivery of **A** short interfering RNA (siRNA) targeting the back-splice junction of circRNAs to induce circRNA cleavage and **B** circRNA expression plasmid to overexpress circRNAs. **C**–**E** Gold nanoparticle-mediated delivery of **C** siRNA targeting the back-splice junction of circRNAs, **D** circRNA expression plasmid, and **E** antisense oligonucleotide (AON) blocking protein interaction site on circRNAs

Several approaches have been developed to study circRNAs (Fig. 2) and target circRNAs for therapeutic purposes in vivo (Fig. 3). In this section, we describe common strategies used to overexpress or knockdown circRNAs. We then discuss approaches that are being explored to improve upon these strategies that may hold therapeutic potential.

### RNA interference-mediated circRNA knockdown

RNA interference (RNAi)-based strategies take advantage of the endogenous RNAi mechanism, in which double stranded RNA (dsRNA) molecules induce post-transcriptional silencing.^127^ Knockdown of circRNAs is typically mediated by RNAi using short interfering RNA (siRNA) or short hairpin RNA (shRNA). siRNAs are 21-23 nt long dsRNA that target circRNAs by complementary pairing and incorporate them into the RNA-induced silencing complex to be cleaved.^32,128^ shRNAs are characterized by their loop and base-paired stems and are subsequently processed into siRNA.^129^ In order to knockdown circRNAs without affecting their corresponding linear mRNA, the back-splice junction unique to circRNAs is usually targeted. Note antisense oligonucleotides (AON) can also target circRNAs through complementary pairing.^130^ Due to their longer length, they are not commonly used to target the back-splice junction to knockdown circRNAs, but they can effectively block protein interaction sites on circRNAs. siRNA and shRNA delivered in lipid-based polymers are currently the most convenient method to knockdown circRNAs in vivo. However, there are several limitations with RNAi molecules, including their rapid degradation by nucleases, low intracellular delivery efficiency, lack of cell-specificity, immunogenicity, and other off-target effects.^27–29,131,132^

### CircRNA expression vectors

CircRNAs are predominantly produced from back-splicing, which can be driven by the pairing of intronic complementary sequences flanking the circularized exons and RBPs. This mechanism is exploited in the design of circRNA expression vectors, which is the standard method to overexpress circRNAs. To date, there have been many vectors reported for circRNA overexpression in cultured cells and animal models.^59,133^ Lentiviral and adenoviral vectors are often used for circRNA overexpression in vivo.^134^ Adeno-associated viruses (AAVs) were used to deliver circRNAs that sponge miRNAs to improve cardiovascular function in transverse aortic constriction (TAC) mice.^135^ AAVs were also used to express circITCH to sponge miR-330-5p, reducing the cardiotoxicity in Doxorubicin-treated mice.^136^

### Synthetic circRNAs

In addition to enhancing circRNA expression using plasmids, direct synthesis and purification of circRNAs can be employed to overexpress circRNAs. There are many different methods to achieve RNA circularization.^137^ Single-stranded linear RNA can be transcribed in vitro or chemically synthesized, then cyclized using splint ligation. This technique produces highly purified circRNA molecules that can then be delivered into target cells.^138^ This technique has been used to engineer an efficient miRNA sponge in vitro.^115^ The potential use of synthetic circRNAs in vivo is limited by difficulties with generating a large amount of circRNAs and unwanted immune system activation.^139^

### Nanoparticle delivery of circRNA-based therapeutics

Nanoparticles can carry therapeutic agents and deliver them to sites affected by disease.^131^ They are currently being investigated for their use in delivering molecules for imaging,^140,141^ therapeutic drugs,^30,131^ and a combination of diagnostic and therapeutic agents.^142^ Many different types of nanoparticle carriers with varying sizes and properties have been engineered. They typically range from a few tenths to a few hundred nm in size.^143^ These carriers can be made of organic materials, like liposomes, polymers, and dendrimers, or inorganic materials like gold and metal oxides.^131,143^ Lipid nanoparticles (LNPs) are the most advanced nanoparticle delivery system.^31^ They can encapsulate siRNA and target specific cells using endogenous or exogenous ligands. LNPs are endocytosed and can subsequently destabilize the endosomal membrane, causing release of siRNA into the cytosol, where they can reach their targets.^31^ Nanoparticles cannot enter the nucleus, so this approach is limited to targeting circRNAs in the cytoplasm. However, this is not a major issue since most circRNAs exert their functions in the cytoplasm.^2^ Importantly, nanoparticle delivery can overcome many limitations of RNAi molecules by protecting them from degradation, facilitating cellular uptake, and preventing immune activation.^30–32,144–146^

The use of nanoparticles as a delivery system has significantly improved the feasibility of circRNA-based therapeutics in vivo. Our lab has been using gold nanoparticles (AuNPs) as a delivery system in animal studies because they are highly stable, pure, and their surface is easy to modify.^144,147^ We delivered AuNPs conjugated with siRNA targeting circDnmt1 or AON targeting binding sites on circDnmt1 for Auf1 and p53 proteins as a therapeutic approach for breast cancer.^15^ We found that both treatments suppressed cellular autophagy and tumor growth and extended the lifespan of mice. In addition, AuNP delivery of AONs blocking binding sites on circCcnb1 for Ccnb1 and Cdk1 inhibited tumor growth and extended mouse viability.^18^ These studies reveal that nanoparticles could be a promising delivery system for circRNA-targeting agents.

Nanoparticles have also been used to deliver circRNA expression plasmids in vivo. In a Dox-induced cardiomyopathy mouse model, AuNP delivery of circAmotl1 plasmids reduced cell apoptosis and improved various measures of heart function.^13^ In a mouse excisional wound model, AuNP delivery of circAmotl1 plasmids promoted skin wound repair.^14^ Furthermore, AuNP delivery of circFoxo3 plasmids increased tumor cell apoptosis and suppressed tumor growth.^17^ More recently, nanoparticle delivery of circEHMT1 plasmids inhibited lung metastasis of breast cancer in mice.^148^ Lastly, AuNP-conjugated circYap plasmids significantly attenuated cardiac fibrosis and improved heart functions in a pressure-overload mouse model.^96^ The delivery efficiency of circRNA nanoparticles is lower than siRNA and AON nanoparticles. An ongoing area of investigation surrounds the optimization of nanoparticle properties to improve their penetrance into and distribution throughout tumors.^149,150^

### Exosome delivery of circRNA-based therapeutics

Exosomes are also currently being explored as delivery vehicles for circRNA-targeting agents and circRNA expression vectors. Exosomes are extracellular vesicles that typically range from 30 to 100 nm in diameter.^151^ They are secreted from and received by many types of cells to facilitate intercellular communication.^151,152^ Exosomes naturally carry a wide variety of molecules, including circRNAs, miRNAs, long noncoding RNAs, proteins, lipids, and DNA fragments.^151,152^ Interestingly, tumor cells have been shown to secrete 10-fold more exosomes than other cells.^153^ Exosomal circRNAs in particular have been found to contribute to cancer progression by promoting cell proliferation, tumor metastasis, and drug resistance.^152^ A recent study found that exosomes from chemoresistant colorectal cancer (CRC) cells contain enriched ciRS-122 circRNA, which sponges miR-122 and upregulates PKM2.^154^ PKM2 in turn promotes glycolysis and ATP production. It was proposed that PKM2 upregulation produces more energy for transporters to expel drugs out of CRC cells. It was shown that chemoresistant CRC cells delivered ciRS-122 via exosomes to nonchemoresistant cells, effectively spreading their drug resistance. This study took advantage of this natural carrier system to deliver siRNA targeting ciRS-122, which increased miR-122 levels and decreased PKM2 levels, ultimately enhancing the sensitivity of CRC cells to oxaliplatin in mice.^154^ Exosomes can also be used to directly deliver circRNA expressing vectors. It was remarkably shown that delivery of engineered rabies virus glycoprotein-circSCMH1-extracellular vesicles facilitated functional recovery after stroke in nonhuman primates.^106^ Similar to nanoparticle delivery systems, exosomes can protect RNAi molecules from degradation and promote cellular uptake without triggering an immune response.^155,156^ Exosomes are likely more biocompatible than synthetic nanoparticles, but this system faces its own manufacturing challenges.^157^

### Conditional circRNA knockout or knockdown

The cre-lox system is widely used to manipulate gene expression in a tissue- or cell type-specific manner.^158^ In a study that investigated the role of circPOK in mesenchymal tumor progression, the cre-lox system was used to conditionally knockout exon 2 of the *Zbtb7a* gene, which gives rise to circPOK and its cognate linear mRNA in mesenchymal cells. Subsequently, POK cDNA was added back to restore linear POK expression.^33^ This strategy can be used to study the function of circRNAs. More recently, the cre-lox system was used in a diabetic mouse model to investigate the role of cZNF532 circRNA in retinal pericyte degeneration and vascular dysfunction.^34^ Cre-dependent shRNA targeting cZNF532 were intraveneously injected into PDGFR-β-cre mice to confer specific knockdown of cZNF532 in pericytes. These results demonstrate that the cre-lox system can be used to knockout or knockdown circRNA in specific cells in vivo.

### CRISPR/Cas9-mediated circRNA knockout or knockdown

The clustered regularly interspaced short palindromic repeats (CRISPR)/CRISPR-associated protein 9 (Cas9) system is a highly specific, efficient, and relatively easy method to edit the genome.^159^ This system uses small guide RNAs (gRNA) to direct the Cas9 nuclease to target and cleave DNA. CRISPR/Cas9 can knockout circRNAs by disrupting the pairing of introns flanking the circularized exons that occurs during circRNA biogenesis. The inverted complementary sequence in the downstream intron of circGCN1L1 was removed in vitro.^160^ As a result, circGCN1L1 levels were undetected. Importantly, its corresponding linear mRNA levels were unaffected. Similarly, CRISPR/Cas9 was used to knockout circHIPK3 in vitro without affecting its linear mRNA, which led to suppressed cell proliferation.^161^ It was found that deleting the downstream inverted repeat ALU element inhibited circHIPK3 formation, but this was surprisingly not the case for the upstream ALU element. It was later determined that deleting the long upstream intron containing ALU repeats also prevented circHIPK3 production.^161^ This demonstrates that it can be difficult to determine which intronic sequences to target due to the complexity of circRNA biogenesis mechanisms. Compared to RNAi-based strategies that directly target the back-splice junction, it is more challenging to use the CRISPR/Cas9 system to try to target intronic sequences involved in circRNA production.

In a more recent study, *CDR1as* locus was removed in vitro. *CDR1as* knockout led to the dysregulation of 353 proteins, many of which were found to interact with each other and play critical roles in cellular pathways.^162^ *CDR1as* locus has also been removed using CRISPR/Cas9 in vivo.^163^ Prior to this deletion, it was verified that the antisense strand to *CDR1as* does not undergo transcription. The results of *CDR1as* knockout did not show any apparent off-target effects. The molecular results suggested specific deregulation of miRNAs known to interact with CDR1as and the upregulation of immediate early genes (IEGs), including targets of those miRNAs. In addition, behavioral studies specifically showed impaired prepulse inhibition, which is associated with the upregulation of IEGs.^163^ Removing the gene locus is a feasible approach for CDR1as because it is circularized very efficiently and does not have a detectable linear counterpart.^49,163^ However, applying this approach to most circRNAs would affect their corresponding linear mRNAs due to the sequence overlap.

CRISPR/Cas9 was also used to knockdown circNfix in vivo by targeting a transcription factor.^92^ This was performed using knock-in loss-of-function mutations introduced in the *Meis1* gene encoding a transcription factor that can bind to a super-enhancer region at the *Nfix* locus to drive circNfix expression. Consequentially, circNfix was knocked down, which led to increased cardiomyocyte proliferation.^92^ This approach may not be generalizable, as this circRNA happens to be regulated by a super-enhancer. Moreover, transcription factors can be involved in the regulation of multiple genes, and targeting them may lead to nonspecific effects.

### CRISPR/Cas13-mediated circRNA knockdown

The CRISPR/Cas13 system specifically targets single-stranded RNA.^164^ Instead of using CRISPR/Cas9 to target the intronic complementary sequences flanking circularized exons or gene loci to knockout circRNAs, CRISPR/Cas13 can directly target the back-splice junction of circRNAs. "Recent studies" (reached similar conclusions...) and move the 2 citations after this sentence as these papers have now been published reached similar conclusions that CRISPR/Cas13 can knockdown circRNAs with high specificity and efficacy. The knockdown efficiency of all available Cas13 family proteins (LwaCas13a, PguCas13b, PspCas13b, RanCas13b, AdmCas13d, EsCas13d, or RfxCas13d) were compared for circPOLR2A and circRTN4 in vitro.^35^ Most of them could knockdown the circRNAs with no detectable effects on their linear counterparts, but RfxCas13d exhibited the highest knockdown efficiency. RfxCas13d was used to target many other circRNAs, and results showed specific and robust knockdown of all circRNAs.^35^ Furthermore, it was found that RfxCas13d exerted a significantly higher circRNA knockdown efficiency compared to position-matched shRNA, with less off-target effects on their corresponding linear mRNA.^35^ In addition, optimal gRNA spacer length and mismatch tolerance of RfxCas13d were investigated. Results showed that circRNA knockdown efficiency was highest when the gRNA spacer, used to target the back-splice junction, was at least 22 nt long.^35^ This was further supported when single or double mismatches were introduced into the gRNAs, and it was found that any mismatch significantly reduced the knockdown efficiency of RfxCas13d, especially in a central seed region extending from the −8 to 8 nt position in a 22 nt long spacer.

Another study evaluated the use of a CRISPR/Cas13d system for circRNA knockdown in vitro. This study evaluated the knockdown of several circRNAs and found that 24–30 nt spacers have comparable knockdown efficiency, with 24 nt spacers showing greater sensitivity to single and double mismatches compared to 30 nt spacers.^36^ It was demonstrated that Cas13d and shRNA could achieve a similar circRNA knockdown efficiency, but Cas13d produced substantially less off-target effects. Furthermore, CRISPR/Cas13d- and shRNA-based circRNA functional screenings were performed, and shRNA screenings were found to result in significantly higher false positive rates.^36^ Altogether, these studies suggest that the CRISPR/Cas13 system could serve as a highly effective tool to directly target circRNAs in a specific and robust manner, and they provide a basis for future in vivo studies.

## Challenges in targeting circRNAs

To date, circRNA-based therapeutic approaches have only been performed in preclinical studies. There are still many obstacles that need to be overcome in order for the therapeutic potential of these approaches to be achieved. Major limitations with these techniques and potential mitigation strategies are outlined in this section.

### Off-target gene silencing

A fundamental concern with RNAi-based strategies is that small molecules like siRNA can potentially induce off-target gene silencing via a miRNA-like effect.^165^ siRNA can target transcripts through partial complementarity, which usually occurs between the 3’UTR of the transcript and seed region of the siRNA.^166,167^ In circRNA knockdown experiments, it is usually verified that the corresponding linear mRNA levels are unaffected. However, off-target effects beyond their linear counterparts are less predictable. Designing siRNA to mitigate off-target effects is an ongoing area of interest for RNAi approaches.^127,157^ The CRISPR/Cas13 system has demonstrated low mismatch tolerance and could knockdown circRNAs with greater specificity than RNAi.^35^ However, whether or not this approach will be effective in vivo remains to be investigated.

### Nonspecific tissue or cell type targeting

Although the majority of circRNAs are expressed in a tissue- or cell type-specific manner, some circRNAs are present in more than one tissue or cell type.^25^ Common strategies used to target circRNAs may cause adverse effects on off-target tissues or cells. Nanoparticle delivery systems have the potential to improve the targeting of therapeutic agents to specific cells.^31,32,168^ Alternatively, this challenge could be avoided in cases where it is possible to target circRNAs with highly specific expression patterns.

### Toxicity of gold nanoparticles

Although AuNPs are convenient for delivering circRNA-targeting agents or circRNA plasmids in animal models, it is unclear how safe they are for clinical use. Previous studies on AuNPs draw inconsistent conclusions about their toxicity.^169^ It has been suggested that its toxic effects are dependent on the size of the particles, with smaller AuNPs causing more harmful effects.^170^ Thus, it is possible that the properties of AuNPs can be fine-tuned to meet safety requirements. Of note, a LNP-siRNA system has already been approved for the treatment of hereditary transthyretin amyloidosis^30^ and could potentially be used to deliver siRNA targeting disease-promoting circRNAs.

### Mis-spliced products

CircRNA overexpression vectors are usually based on the pairing of intronic complementary sequences. This system can lead to mis-splicing of linear RNAs or circRNAs. The mis-spliced byproducts can cause nonspecific and potentially deleterious effects. currently, there are still no vectors that can generate target circRNAs without mis-spliced products. Highly purified circRNA molecules synthesized in vitro could potentially be used to overcome the shortcomings of circRNA overexpression vectors. However, inherent problems with large-scale synthesis may limit the therapeutic potential of synthetic circRNAs.

### Synthetic circRNA immunogenicity

In addition, synthetic circRNAs can induce immune system activation in vivo.^171^ It was suggested that foreign circRNAs are distinguished from endogenous circRNAs based on their lack of the m^6^A modification.^138^ Strategies are currently being explored to reduce synthetic circRNA immunogenicity, including introducing chemical modifications and coating them in RBPs.^139^

## Perspective

In recent years, many studies have contributed to our increasing understanding of circRNA functions and their important roles in diseases. Due to their stability and tissue- or cell type-specific expression, circRNAs have emerged as promising therapeutic targets. Therefore, it is necessary to develop tools that can effectively target circRNAs. In this review, we presented several recent developments, including nanoparticle- and exosome-mediated delivery of circRNA-based therapeutics, synthetic circRNAs, conditional and CRISPR-Cas9-mediated circRNA knockout or knockdown, CRISPR/Cas9-mediated circRNA knockout, and CRISPR/Cas13-mediated circRNA knockdown. Future investigations should further examine the safety and efficacy of nanoparticles and exosomes as delivery vehicles for circRNA-based therapeutics in vivo. Importantly, the CRISPR/Cas13 system appears to be a promising approach to knockdown circRNAs with great specificity and efficiency and warrants investigation in vivo. Out of all the approaches discussed, siRNA technology is currently the most feasible. siRNA nanoparticles have been previously approved for therapeutic treatment and are currently under investigation in clinical trials. This approach appears promising for clinical application. If a circRNA is oncogenic, siRNA targeting the junction sequence could downregulate the oncogenic circRNA to intervene in cancer progression. If a circRNA induces cardiac fibrosis and impairs heart function, silencing the circRNA using siRNA could provide beneficial outcomes for patients with heart disease. On the other hand, delivery of synthetic circRNAs may be difficult due to the limited efficiency of delivering circRNAs of a large size. Moreover, it is not a realistic approach to generate a large amount of circRNAs. CRISPR/Cas9 and CRISPR/Cas13 have the potential to knockout or knockdown particular circRNAs and could have clinical applications in the future. While these approaches have been shown to be effective in targeting circRNAs, the expression vehicle could produce unknown side effects. Similarly, circRNA expression viral vectors could also generate unexpected side effects. Therefore, many obstacles still remain in this field. In the coming years, studies that expand our knowledge on circRNA functional mechanisms and further the development of specific and effective approaches to target circRNAs in vivo will be key in advancing the clinical potential of circRNA-based therapeutics.
